# Differentiation of Human Induced-Pluripotent Stem Cells into Smooth-Muscle Cells: Two Novel Protocols

**DOI:** 10.1371/journal.pone.0147155

**Published:** 2016-01-15

**Authors:** Libang Yang, Zhaohui Geng, Thomas Nickel, Caitlin Johnson, Lin Gao, James Dutton, Cody Hou, Jianyi Zhang

**Affiliations:** 1 Division of Cardiology, Department of Medicine, University of Minnesota Medical School, Minneapolis, Minnesota, United States of America; 2 Stem Cell Institute, University of Minnesota Medical School, Minneapolis, Minnesota, United States of America; 3 Department of Biomedical Engineering, University of Minnesota, Minneapolis, Minnesota, United States of America; 4 Department of Integrative Biology and Physiology, University of Minnesota, Minneapolis, Minnesota, United States of America; 5 Department of Biomedical Engineering, School of Medicine, School of Engineering, The University of Alabama at Birmingham, Birmingham, Alabama, United States of America; Northwestern University, UNITED STATES

## Abstract

Conventional protocols for differentiating human induced-pluripotent stem cells (hiPSCs) into smooth-muscle cells (SMCs) can be inefficient and generally fail to yield cells with a specific SMC phenotype (i.e., contractile or synthetic SMCs). Here, we present two novel hiPSC-SMC differentiation protocols that yield SMCs with predominantly contractile or synthetic phenotypes. Flow cytometry analyses of smooth-muscle actin (SMA) expression indicated that ~45% of the cells obtained with each protocol assumed an SMC phenotype, and that the populations could be purified to ~95% via metabolic selection. Assessments of cellular mRNA and/or protein levels indicated that SMA, myosin heavy chain II, collagen 1, calponin, transgelin, connexin 43, and vimentin expression in the SMCs obtained via the Contractile SMC protocol and in SMCs differentiated via a traditional protocol were similar, while SMCs produced via the Sythetic SMC protocol expressed less calponin, more collagen 1, and more connexin 43. Differences were also observed in functional assessments of the two SMC populations: the two-dimensional surface area of Contractile SMCs declined more extensively (to 12% versus 44% of original size) in response to carbachol treatment, while quantification of cell migration and proliferation were greater in Synthetic SMCs. Collectively, these data demonstrate that our novel differentiation protocols can efficiently generate SMCs from hiPSCs.

## Introduction

Human induced-pluripotent stem cells (hiPSCs) can provide a theoretically unlimited number of terminally differentiated cells for use in tissue engineering, drug development, and autologous cell therapy; however, their utility will remain limited (particularly for clinical applications) until efficient, standardized differentiation protocols are developed to satisfy the requirements of Good Manufacturing Practice. Protocols for differentiating hiPSCs into endothelial cells (hiPSC-ECs) [1] and cardiomyocytes (hiPSC-CMs) [2] have recently been improved, but conventional methods for generating hiPSC-derived smooth-muscle cells (hiPSC-SMCs) can take longer than four weeks [3] and may rely on co-culturing with feeder cells, which can lead to xenogenic contamination [4].

Because smooth muscle cells (SMCs) develop from a wide range of embryonic tissues, including the neural crest [5], the paraxial/somatic mesoderm [6], the lateral plate mesoderm [7], and the secondary heart field [8], many hiPSC-SMC differentiation protocols direct the cells toward an intermediate, origin-specific lineage [9, 10] before inducing the terminal SMC phenotype. Furthermore, somatic SMCs display a wide range of morphological and functional characteristics that are best described as a spectrum bounded by predominantly synthetic and contractile phenotypes [11]. Here, we present two hiPSC-SMC differentiation protocols. Both protocols begin by using a GSK inhibitor (CHIR99021) and bone morphogenic protein 4 (BMP-4) to direct the hiPSCs toward the mesodermal lineage; then, Synthetic hiPSC-SMCs are produced by culturing the cells with vascular endothelial growth factor (VEGF) and fibroblast growth factor (FGF), or the Contractile hiPSC-SMC phenotype is induced with varying combinations of platelet-derived growth factor (PDGF), transforming growth factor (TGF), and FGF. Each protocol can be completed in two to three weeks and includes a 4- to 6-day selection period, which yields SMC populations that are ~95% pure and remain phenotypically stable for at least 20 generations.

## Methods

### Cell lines

The differentiation protocols were tested with hiPSCs that had been reprogrammed from human cardiac fibroblasts [12] or from human dermal fibroblasts [1] (GriPS, kindly provided by Dr. James Dutton, University of Minnesota, USA) and with H9 embryonic stem cells [13] (ESCs) (kindly provided by Dr James Thomson, University of Wisconsin, Madison, USA). Control assessments were performed with hiPSC-SMCs that had been differentiated via a conventional protocol [14] and in primary human aortic SMCs (HA-SMCs) (Life Technologies Corporation, Grand Island, NY, USA).

### Synthetic and contractile hiPSC-SMC differentiation protocols

**Fig 1:**
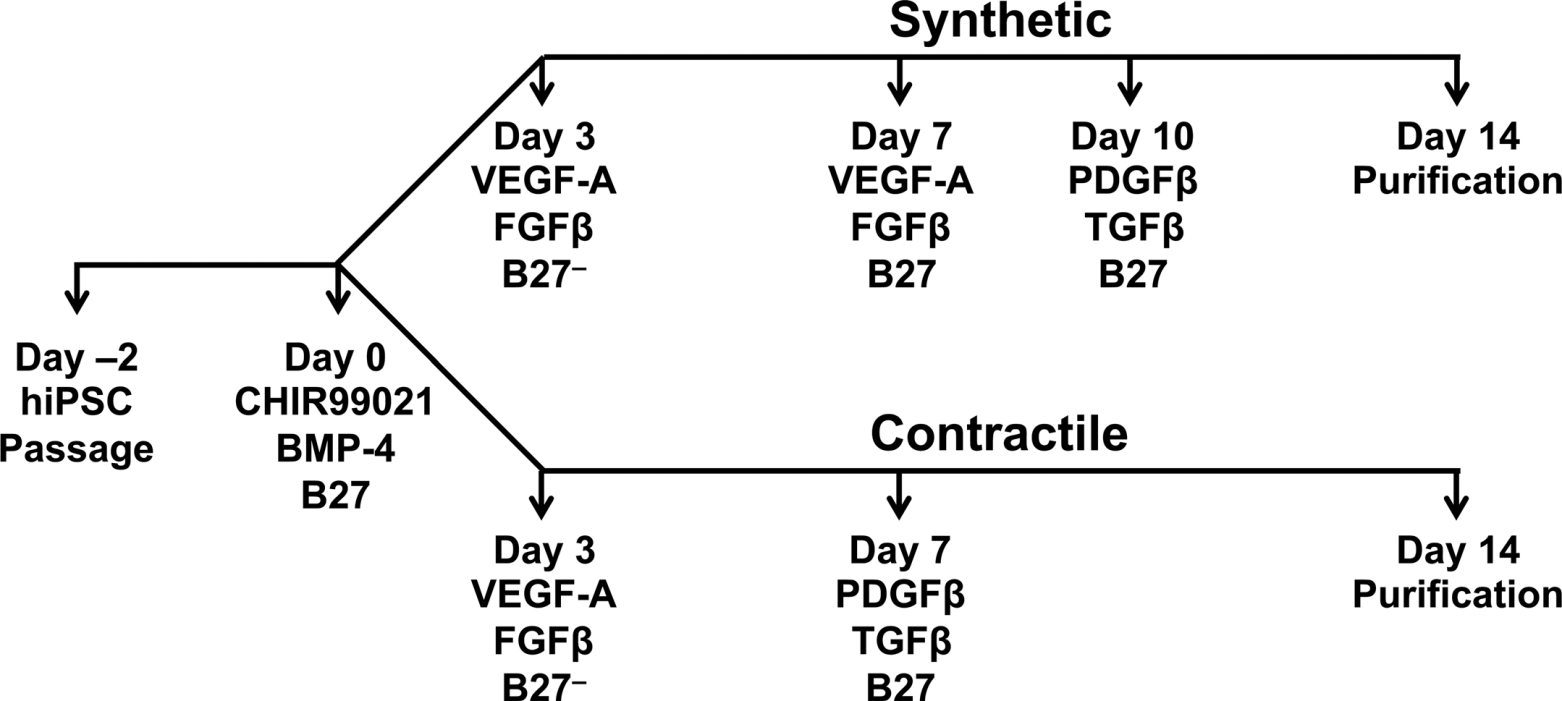
Chronological summary of the hiPSC-SMC differentiation protocols. hiPSCs and ESCs were cultured in mTeSR^TM^ medium on Matrigel-coated plates, with daily medium changes, until confluent (~2 days); then, differentiation into mesodermal-lineage cells was initiated on Day 0 by culturing the cells with CHIR99021 and BMP-4 in RPMI1640 medium and 2% B27. Differentiation into Synthetic SMCs or Contractile SMCs began on Day 3. Synthetic SMCs were produced by culturing the cells with VEGF-A and FGFβ in RPMI1640 medium and 2% B27 minus insulin (B27^–^) from Day 3 to Day 7, with VEGF-A and FGFβ in RPMI1640 and 2% B27 (with insulin) from Day 7 to Day 10, and with PDGFβ and TGFβ in RPMI1640 and 2% B27 from Day 10 to Day 14. Contractile SMCs were produced by culturing the cells with VEGF-A and FGFβ in RPMI1640 and 2% B27^–^ from Day 3 to Day 7, and with PDGFβ and TGFβ in RPMI1640 and 2% B27 from Day 7 to Day 14. Purification was performed by maintaining the differentiated cells in 4 mM lactate RPMI1640 metabolic medium for 4 to 6 days.

hiPSCs and ESCs were cultured in mTeSR^TM^ medium on Matrigel-coated plates, with daily medium changes, until confluent (~2 days); then, differentiation into mesodermal-lineage cells was initiated on Day 0 by culturing the cells with CHIR99021 (5 μM) and BMP-4 (10 ng/mL) in RPMI1640 medium and 2% B27. Differentiation into Synthetic SMCs or Contractile SMCs began on Day 3. Synthetic SMCs were produced by culturing the cells with 25 ng/mL VEGF-A and FGFβ in RPMI1640 and 2% B27 minus insulin from Day 3 to Day 7, with 25 ng/mL VEGF-A and FGFβ in RPMI1640 and 2% B27 from Day 7 to Day 9, and with 10 ng/mL PDGFβ and 3 ng/mL TGFβ in RPMI1640 and 2% B27 from Day 10 to Day 14. Contractile SMCs were produced by culturing the cells with 25 ng/mL VEGF-A and FGFβ in RPMI1640 and 2% B27 minus insulin from Day 3 to Day 7, and with 5 ng/mL PDGFβ and 2.5 ng/mL TGFβ in RPMI1640 and 2% B27 from Day 7 to Day 14. The differentiated cells were enriched for SMCs by maintaining them in 4 mM lactate RPMI1640 metabolic medium for 4 to 6 days (Fig 1).

### Quantitative real time polymerase chain reaction (QRT-PCR)

Total RNA was extracted with RNeasy Mini as directed by the manufacturer’s instructions (QIAGEN). cDNA was prepared with a Maxima First Strand cDNA Synthesis Kit (Thermal Scientific Inc), and the QRTPCR mixtures were prepared with SYBR Green PCR Master Mix (Thermal Scientific Inc). QRTPCR reactions were performed on a 7500 Fast Real-time PCR System (Applied Biosystems) and by using the Quantitation-comparative C_T_ setting. The QRTPCR thermal cycling program included 40 cycles, and each cycle consisted of enzyme activation for 2 min at 95°C, denaturation for 30 sec at 95°C, annealing for 30 sec at 60°C, and extension for 30 sec at 70°C; primer sequences are listed in Table 1. Duplicate measurements were performed for each analysis and were normalized to the endogenous level of glyceraldehyde-3-phosphate dehydrogenase (GAPDH) mRNA.

**Table 1:** QRT-PCR Primer Sequences.

Gene	Primer Sequences	
**Alpha SMA 2**	Forward:	GAT CTG GCA CCA CTC TTT CTA C
	Reverse:	CAG GCA ACT CGT AAC TCT TCT C
**Calponin**	Forward:	ATG TCC TCT GCT CAC TTC AAC
	Reverse:	CAC GTT CAC CTT GTT TCC TTT C
**Myosin heavy chain 11**	Forward:	AGG CGA ACC TAG ACA AGA ATA AG
	Reverse:	CTG GAT GTT GAG AGT GGA GAT G
**Transgelin**	Forward:	GAA GAA AGC CCA GGA GCA TAA
	Reverse:	CCA GGA TGA GAG GAA CAG TAG A
**VE cadherin**	Forward:	GAA ACA GAG CCC AGG TCA TTA
	Reverse:	GAT GGT GAG GAT GCA GAG TAA G

Alpha SMA 2, alpha smooth-muscle actin 2; VE cadherin, vascular endothelial cadherin.

### Immunofluorescent imaging and flow cytometry

For immunofluorescent imaging, adherent cells were fixed, permeabilized, blocked with 5% donkey serum, and incubated with primary antibodies at 4°C overnight; then, the primary antibodies were labeled with fluorescent secondary antibodies (Jackson Lab, USA) (Table 2), cell nuclei were labeled with DAPI, and the cells were imaged under an Olympus 200M microscope. For flow-cytometry analyses, cells were fixed with 4% paraformaldehyde for 10 min and incubated with UltraV (Thermo Scientific, USA) block for 7 min at room temperature; then, the cells were labeled with primary phycoerythrin (PE)-conjugated or allophycocyanin (APC)-conjugated anti-SMA antibodies and with isotype-control antibodies (BD Pharmingen, USA) for 30 min at 4°C in phosphate-buffered saline (PBS) and 2% fetal bovine serum (FBS), washed with 2% FBS/PBS, and re-suspended in 0.3 mL 2% FBS/PBS containing 5 μL of propidium iodide (10 μg/mL). Flow-cytometry analyses were performed with a FACS Aria instrument (BD Biosciences, USA), and control assessments were performed with undifferentiated hiPSCs.

**Table 2:** Antibodies.

Antibody	Isotype/Source/Catalog Number/Clone	Concentration
αSMA	Mouse IgG/Sigma/0837M4778V/Mab	1:150
Connexin 43	Rabbit IgG/Millipore/AB1728/Polyclonal	1:100
Collagen I	Mouse IgG/ Millipore /MAB3391/Mab	1:150
Calponin	Mouse IgG/ Transduction Labs/8592591550/Mab	1:100
Vimentin	Goat IgG/R&D systems/AF2105	1:100
Secondary antibody	FITC Donkey Anti-mouse IgG/703-099-155	1:100
Secondary antibody	FITC Donkey Anti-rabbit IgG/711-095-152	1:100
Secondary antibody	FITC Donkey Anti-goat IgG/705-095-147	1:100
Secondary antibody	TRITC Donkey Anti-mouse IgG/Jackson Lab/712-025-150	1:100
Secondary antibody	TRITC Donkey Anti-rabbit IgG/ Jackson Lab/711-026-152	1:100
Secondary antibody	TRITC Donkey Anti-goat IgG/ Jackson Lab/705-025-147	1:100
Secondary antibody	Alexa-Fluor647 Donkey Anti-mouse IgG/ Jackson Lab/ Jackson Lab/715-495-140	1:100
Secondary antibody	Alexa-Fluor647 Donkey Anti-rabbit IgG/ Jackson Lab/711-605-152	1:100
Secondary antibody	Alexa-Fluor647 Donkey Anti-goat IgG/ Jackson Lab/705-605-147	1:100
Secondary antibody	FITC Donkey Anti-mouse IgM/ Jackson Lab/715-026-020	1:100
Secondary antibody	TRITC Donkey Anti-mouse IgM/ Jackson Lab/715-096-020	1:100

### Cell migration

Cells were cultured in 6-well plates (4×10^5^ cells/well) overnight at 37°C in Dulbeco’s Modified Eagle Medium (DMEM) containing 10% FBS under a water-saturated, 5% CO_2_ atmosphere; then, the cultures were scratched with a 200-μL pipette tip and the medium was changed to DMEM containing 1% FBS. Images were taken 10 hours later, and cell migration was quantified as the number of cells present in the scratched region.

### Contraction

#### Cell contraction test

Cells were seeded onto a gelatin-coated 6-well plate (2×10^5^ cells/well), partially detached by incubating them with a non-enzymatic dissociation buffer (Versene, Invitrogen, USA), and then treated with 10 μM carbachol for 5 min. Images were obtained both 0 and 5 min after carbachol treatment, and contraction was evaluated by using CellC Software (Department of Signal Processing, Tampere University of Technology, https://sites.google.com/site/cellcsoftware/download) to measure the cells’ cross-sectional surface areas.

#### Gel contraction test

SMCs (1×10^6^) were suspended in 250 μL of an 8 mg/mL fibrinogen solution; then, the cell-containing fibrinogen solution was mixed with 250 μL of a 5 U/mL thrombin solution in one well of a 24-well plate and incubated at 37°C. Ten min later (after the mixture had formed a semi-solid gel) the gels were transferred to a 6-well plate and cultured with 300 U/mL aprotinin and 10 μM Rho kinase (ROCK) inhibitor (Y-27632; Millipore, USA) in 2 mL DMEM containing 5% PBS. Gel sizes were measured at 24-hour intervals over 3 days.

### Cell proliferation

SMCs (1×10^6^ cells/mL in RPMI1640) were serially diluted in a 1:2 ratio, and 100 μL of solution for each cell concentration was added to the wells of a 96-well plate. The cells were cultured with PDGFβ and TGFβ in DMEM containing F12 and 5% FBS for 2 hours under 5% CO_2_; then, 20 μL of One Solution Cell Proliferation Assay reagent (Promega, USA) was added to the well, and the cells were cultured under the same conditions for another 90 min. Proliferation was quantified by measuring optical density at 490 nm with an ELISA reader (Energy H_2_, Bio-Tek instrument, USA) both before (i.e., at 0 min) and after the 90-min culture period; measurements were blanked with the One Solution Cell Proliferation Assay reagent, and the measurement at 0 min was used to define the baseline.

### Statistics

Data are presented as mean±standard deviation (SD) and were evaluated for significance via the student T test (unpaired, 2 tailed); the Bonferroni correction was used when more than two groups were being compared. Statistical analyses were performed with SPSS software (version 20.0; IBM, Armonk, New York, USA) and a p-value of less than 0.05 was considered significant.

## Results

### Differentiation of hiPSCs into hiPSC-SMCs

**Fig 2:**
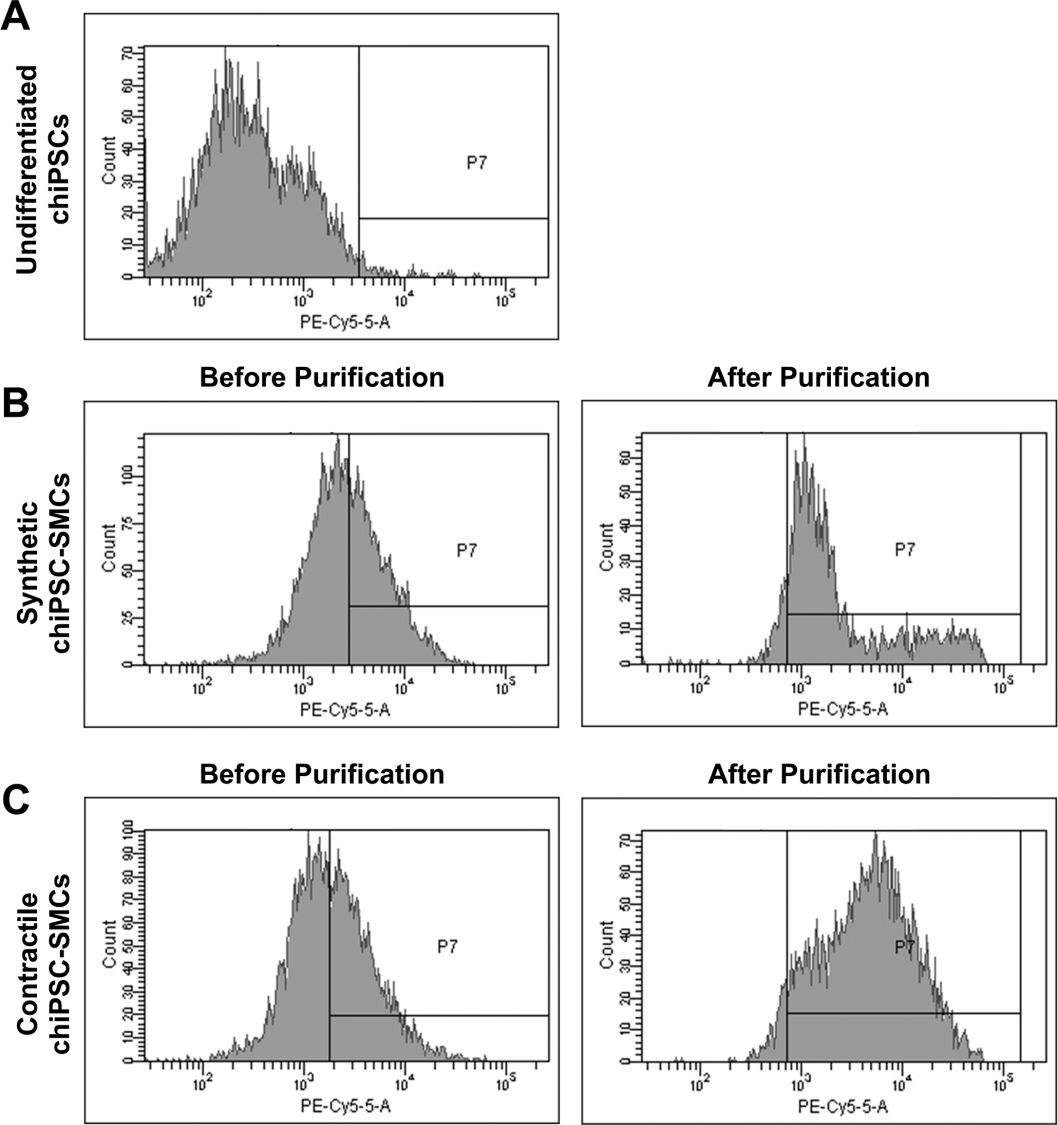
Efficiency of hiPSC-SMC differentiation. The efficiency of the differentiation protocols was evaluated via flow cytometry analyses of SMA expression in (A) undifferentiated cardiac-lineage hiPSCs (chiPSCs) and in (B-C) chiPSC-SMCs obtained via the (B) Synthetic and (C) Contractile SMC differentiation protocols before and after purification.

The efficiency of our differentiation protocols was evaluated in cardiac-lineage hiPSCs (chiPSCs), dermal-lineage hiPSCs (dhiPSCs), and embryonic stem cells (ESCs). The cells were incubated on Matrigel-coated plates for two days until they reached near-complete confluence. Differentiation into the mesodermal lineage was initiated on Day 0 and produced cultures of uniform morphology with a small amount of cell death (10%~15%) that can likely be attributed to CHIR99021 toxicity. Induction of the SMC phenotypes began on Day 3, and then the differentiated cells were purified by maintaining them in metabolic medium for 4 to 6 days beginning on Day 14 (Fig 1). Before purification, flow cytometry analyses indicated that SMA was expressed by ~45% of chiPSC-derived SMCs (Synthetic: 46%, Contractile: 44.5%; n = 6) (Fig 2), by 35% (Contractile; n = 3) to 75% (Synthetic; n = 3) of dhiPSC-SMCs, and by ~80% (Synthetic: 83%, Contractile: 78%; n = 3) of hESC-derived SMCs. The proportion of SMA^+^ cells increased to ~95% after purification (chiPSC-SMCs, Synthetic: 95.1%, Contractile: 94.5%; n = 6. dhiPSC-SMCs, Synthetic: 97%, Contractile: 96.4%; n = 3. hESC-SMCs, Synthetic: 98%, Contractile: 98.6%; n = 3).

### Characterization of Synthetic and Contractile hiPSC-SMCs

**Fig 3:**
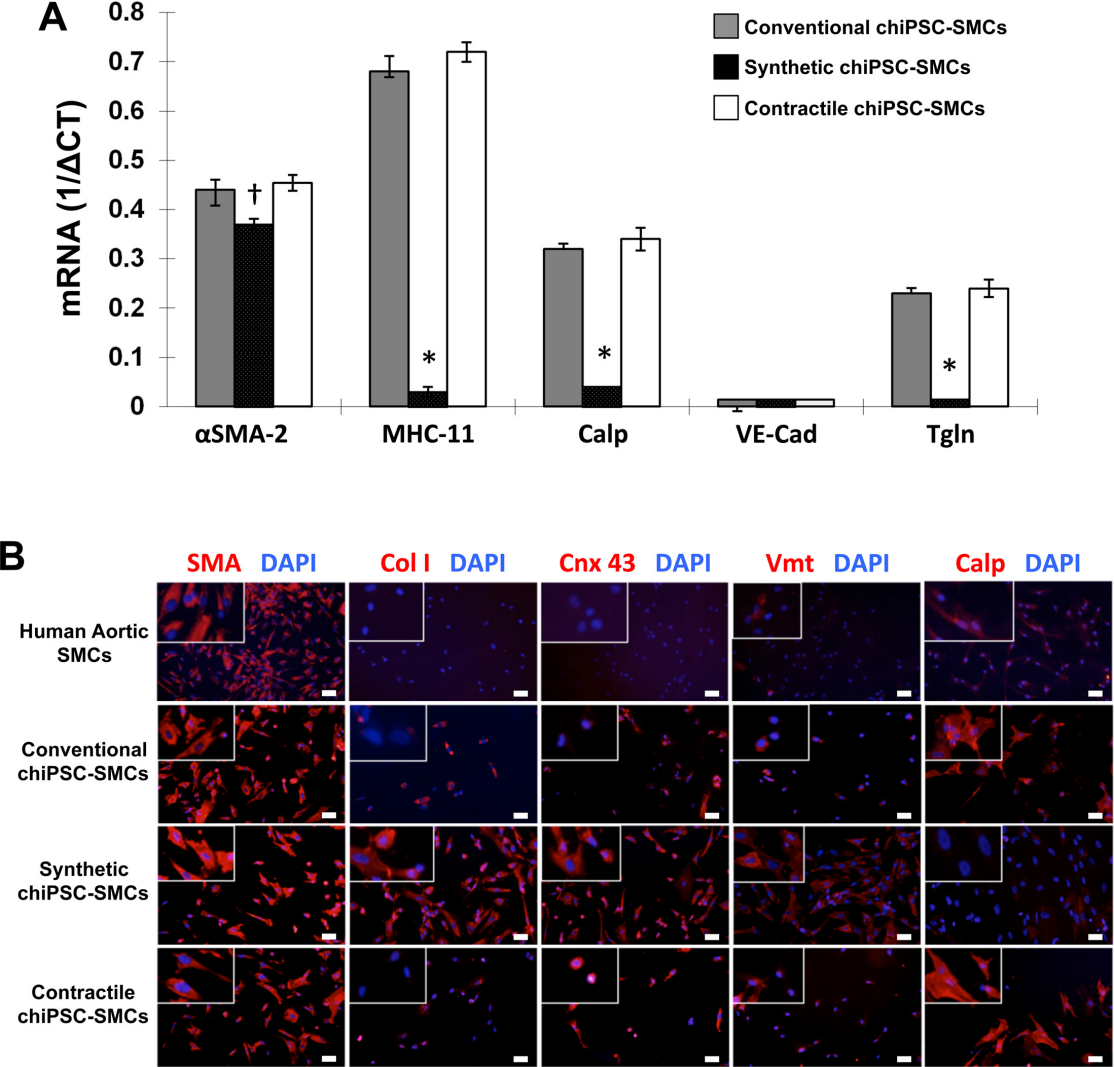
hiPSC-SMC marker expression. (A) mRNA levels of the SMC markers alpha smooth-muscle actin 2 (αSMA-2), smooth muscle myosin heavy chain 11 (MHC-11), calponin (Calp), vascular-endothelial cadherin (VE-Cad), and transgelin (Tgln) were evaluated via quantitative RT-PCR and normalized to endogenous GAPDH mRNA levels (*p<0.01 vs Conventional or Contractile chiPSC-SMCs, ^†^p<0.05 vs Conventional chiPSC-SMCs, ^†^p<0.01 vs Contractile chiPSC-SMCs). (B) Smooth-muscle actin (SMA), collagen I (Col I), connexin 43 (Cnx 43), vimentin (Vmt), and calponin (Calp) protein expression (red) was detected via immunofluorescent staining in human aortic SMCs, in chiPSC-SMCs that were obtained via a conventional differentiation protocol, and in chiPSC-SMCs obtained via our Synthetic or Contractile hiPSC-SMC differentiation protocols; nuclei were counterstained with DAPI (blue) (bar = 100 μm).

**Fig 4:**
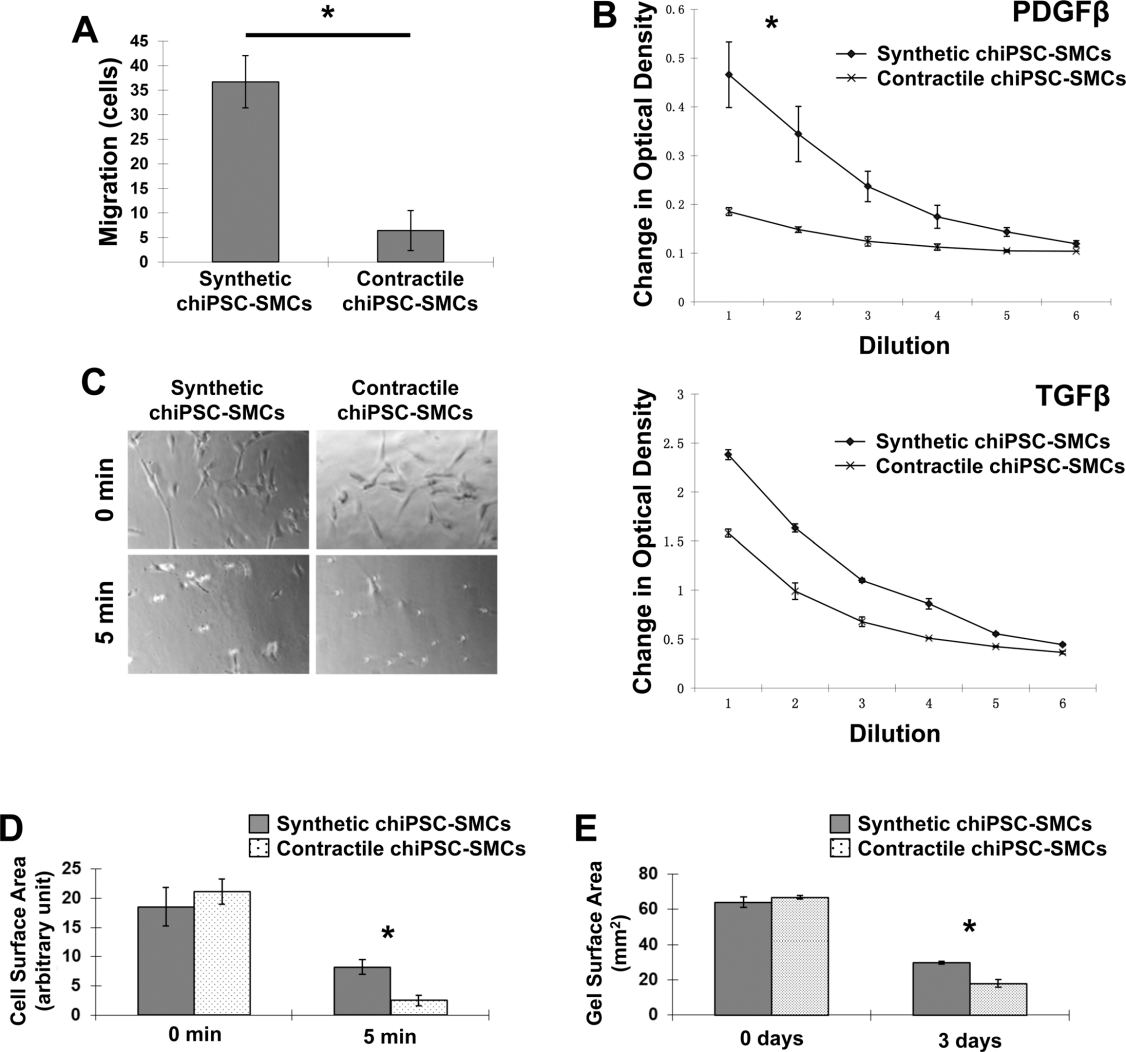
hiPSC-SMC functional assessments. (A) 4×10^5^ Synthetic or Contractile chiPSC-SMCs were cultured on gelatin-coated plates for 24 hours; then, the plate was scratched with a 200-μL pipette tip, and images of the scratched area were obtained 0 and 10 hours later. Migration was quantified by counting the number of cells that had migrated into the scratched area (*p<0.01). (B) 1×10^6^/mL Synthetic or Contractile chiPSC-SMCs were suspended in 100 μL of RPMI1640 and cultured in the presence of PDGFβ or TGFβ for 90 min; then, the solutions were serially diluted in half six times, and cell concentrations were evaluated via optical density measurements at 490 nm (*p<0.01). (C) 2×10^5^ Synthetic or Contractile chiPSC-SMCs were cultured on gelatin-coated plates for 24 hours; then, the cells were treated with carbachol to induce contraction, and images were obtained 0 and 5 min later. (D) Contraction was evaluated by calculating the mean cell surface area at each of the two time points (*p<0.01). (E) 1×10^6^ Synthetic or Contractile chiPSC-SMCs were suspended in a fibrinogen gel; then, the gels were cultured with aprotinin and Rho kinase inhibitor, and the surface area of the gels was measured 0 and 3 days later (*p<0.01).

The specificity of the two hiPSC-SMC differentiation protocols was evaluated by comparing the expression of cell-specific markers in chiPSC-derived Synthetic and Contractile SMCs, in hiPSC-SMCs that were generated via conventional differentiation methods, and in primary human aortic SMCs (HA-SMCs). Myosin heavy chain 11, calponin, and transgelin mRNA levels in conventional hiPSC-SMCs and Contractile chiPSC-SMCs were similar and significantly higher than in Synthetic chiPSC-SMCs (Fig 3A), but Synthetic chiPSC-SMCs were more likely to express collagen 1, connexin 43, or vimentin (Fig 3B). Assessments of cell migration (Fig 4A) and proliferation (Fig 4B) were also significantly greater in Synthetic chiPSC-SMCs than in Contractile chiPSC-SMCs, but the two-dimensional surface area of Contractile chiPSC-SMCs declined more extensively in response to carbachol treatment (Fig 4C and 4D). Gels containing Contractile chiPSC-SMCs also contracted to 27% of their original size, compared to 46% for gels that contained Synthetic chiPSC-SMCs (Fig 4E).

## Discussion

Although the contractile activity of SMCs may be their most prominent characteristic, SMCs also contribute to a variety of other physiological activities, including the growth and remodeling of vessels in response to vascular injury, exercise, or pregnancy [15]. The functional diversity of SMCs is accompanied by considerable phenotypic diversity, ranging from contractile cells at one extreme to predominantly synthetic cells at the other. These two archetypal SMC phenotypes differ substantially in morphology, marker expression, and activity, including their rates of proliferation and migration. Thus, the utility of hiPSC-derived SMCs for a particular application may depend on the specific phenotype generated. For example, pharmacological studies of vasoconstriction may be best performed with populations of primarily contractile SMCs, while tissue engineering [9, 10, 16–19] and cell therapy could benefit from the inclusion of synthetic SMCs, which are more proliferative and produce larger amounts of extracellular matrix material [20].

A number of highly efficient hiPSC-SMC differentiation protocols have been developed [14, 21, 22], but methods for specifying the contractile or synthetic SMC phenotype have been unavailable until recently [23, 24]. The results presented here indicate that our novel differentiation protocols effectively direct hiPSCs toward either a contractile or synthetic SMC phenotype, and that the SMC populations can be purified to ~95% via metabolic selection. Furthermore, the risk of xenogenic contamination is minimized, because the differentiation and purification procedures do not require exposure to feeder cells, and the entire protocol (both differentiation and purification) can be completed in just 2–3 weeks. The cells can also be quickly expanded in DMEM with 5 ng/mL FGF and 5% serum or Medium 231 (Life Technologies, USA), and the SMC phenotype remains stable for at least four months or 20 generations when the cells are maintained in 5% FBS or RPMI-1640 medium with B27. Proliferation can be halted by limiting the serum concentration or by reducing the cell density to ~10% or less (e.g., 5×10^4^ cells/well in a 6-well plate).

The predominant phenotype of an hiPSC-SMC population can be characterized by evaluating the expression of a panel of cell-surface markers [23]. Marker expression in SMCs generated via our Contractile SMC differentiation protocol was similar to the expression in conventionally produced hiPSC-SMCs, while the synthetic SMC differentiation protocol yielded cells that produced more of the extracellular matrix proteins collagen 1 and connexin 43. These observations are consistent with the results from studies with primary SMCs that have been isolated from swine coronary arteries [25, 26], although the pattern of gene expression appears to vary depending on whether the cells are studied *in vivo* or *in vitro* [11, 27]. Furthermore, some evidence suggests that marker expression in SMCs derived from hiPSCs and hESCs, or from hiPSCs that were generated from different somatic tissues, can also differ [23, 24]. Thus, the efficiency of our hiPSC-SMC differentiation protocols and subsequent function of the differentiated cells may depend on which hiPSC (or hESC) line is used.

In conclusion, we have developed two novel, highly efficient differentiation protocols that can be used to generate contractile or synthetic SMCs from hiPSCs in just 2–3 weeks while minimizing the risk for xenogenic contamination.
